# Promoting Tobacco Cessation in a Community-Based Women’s Health Centre

**DOI:** 10.4172/2167-0420.1000225

**Published:** 2015-02-25

**Authors:** Annamaria Masucci Twarozek, Thomas Eggert, Zachary G Puca, Nefertiti DuPont, Deborah O Erwin, Chester H Fox, Martin C Mahoney

**Affiliations:** 1 Roswell Park Cancer Institute, Elm & Carlton Streets, Buffalo, New York; 2 State University of New York at Buffalo, School of Medicine and Biomedical Sciences, Buffalo, New York; 3 Doctor of Oncology, Roswell Park Cancer Institute, Elm & Carlton Streets, Buffalo, New York; 4 Director, Office of Cancer Health Disparities Research, Roswell Park Cancer Institute, Elm & Carlton Streets, Buffalo, New York; 5 Department of Family Medicine, State University of New York at Buffalo, 77 Goodell Street, Buffalo New York; 6 Departments of Medicine & Health Behaviour, Roswell Park Cancer Institute, Elm & Carlton Streets, Buffalo, New York

**Keywords:** Smoking cessation, Women’s health, Adult, Female, Tobacco use disorder, Urban, Community-based clinics

## Abstract

**Objective:**

This report assesses the impact of a series of education sessions delivered to office staff on the delivery of smoking cessation services among patients seeking care at a community-based women’s health center.

**Methods:**

A quasi-experimental design was used to examine the delivery of smoking cessation services to patients in a medical office before and after office staff attended a series of 3 educational sessions intended to increase their knowledge and self-efficacy to address cessation. Delivery of smoking cessation services was documented through a systematic review of medical records using a structured abstraction form.

**Results:**

While nearly all smokers (93%) were asked about smoking status at their last office visit, few smokers at baseline or follow-up were assessed for interest in setting a quit date or offered pharmacotherapy. Referrals to the smokers quit line increased from <1% at baseline to 8% at follow-up (p<0.001) and “any assistance” also showed a modest but significant increase (<1% baseline, 9% follow-up, p<0.001).

**Conclusion:**

This evaluation failed to identify clinical meaningful changes in the delivery of smoking cessation services in this women’s health office before and after completion of a series of educational interventions for office staff. It is anticipated that the implementation of patient centered medical homes, and EMR systems, will help to enhance the delivery of smoking cessation services to women seeking medical care.

## Introduction

Smoking rates continue to decline with 18.1% of adults age 18 or older identified as currently smoking cigarettes in the United States in 2012, compared to 20.9% of adults who were smokers in 2005 [1]. Even with this decrease in use, tobacco use remains as the leading cause of preventable death in the United States, accounting for nearly 480,000 deaths per year [2]. Smokers are at an increased risk of morbidity and mortality from an array of smoking associated health outcomes including cardiovascular disease, pulmonary disease, lung diseases and cancer [3,4]. Lifelong smoking reduces overall life expectancy by about 10 years compared to never smokers [4].

Education and income are inversely associated with smoking behaviours. Persons with limited educational attainment (<=high school diploma, 45.3% smoking rate) exhibit the highest rates of tobacco use compared to those with associate (17.9%), bachelor (9.1%) and graduate degrees (5.9%). Also, persons living below the poverty level have a smoking rate of 27.9% compared to 17.0% for persons at or above the poverty level [1]. In addition, Medicaid populations are more than twice as likely to report current smoking compared to the general population [5].

Tobacco cessation counselling increases the likelihood of successfully quitting, more intensive counselling (e.g., in office counselling of >10 minutes or 4+ sessions) increases effectiveness by about 2-fold [3]. However, combining counselling with the use of effective pharmacotherapies increases the chances of successful quitting to about 25% to 30% [3]. In contrast, smokers who make an unaided quit attempt, without counselling and a cessation medication, have a 4–7% chance of being successful [3].

In 2010, 68.8% of US adult smokers reported they would like to completely quit; including 52.4% who made a quit attempt during that year [6]. Most smokers (54%) report being advised to quit by a health care provider. Advice to quit from any health care professional does vary by gender, age group, race/ethnicity, marital status, geographic region and health insurance with males, persons 18–24 years, non-Hispanic blacks and Hispanics, never married, residents of the South minorities and persons without health insurance are all less likely to receive cessation advice; cessation advice was not associated with income level or educational attainment [7].

While all visits provide an occasion to assess smoking status, women’s well visits provide a unique opportunity for physicians and medical office staff to promote smoking cessation since smoking contributes to the persistence of Human Papilloma Virus (HPV) infections which can lead to abnormal pap smears, cervical dysplasia and cervical cancer. The purpose of this report is to assess the impact of a series of education sessions delivered to office staff on the delivery of smoking cessation services among patients seeking care at a community-based women’s health center.

## Methods

### Study design

This study utilized a quasi-experimental design to examine pre-versus post- intervention assessments of the delivery of smoking cessation services to patients based upon a review of medical records.

### Study population/intervention/office Setting

The study population included staff in the medical office, included 3 advanced practitioners (nurse practitioners and physician assistants) and 5 nursing staff (registered nurses and medical assistants). The intervention consisted of a series of three mandatory educational sessions, delivered monthly over 3 months, to office staff (n=7–8 persons per session) during lunch breaks. The sessions addressed 1) the systematic identification of smokers, 2) use of cessation pharmacotherapy and 3) motivational interviewing/counselling skills; each session last 45–50 minutes. These topics were identified based upon formative research conducted with office staff (unpublished data) as part of on-going community-based collaborative activities.

This medical office is located in a medically underserved community within Buffalo, New York. Buffalo ranks as one of poorest cities in the country with a poverty rate of 31.4% compared to a national rate of 14.5% [8]. African Americans account for 39% of the 260,000 residents in the city of Buffalo compared to 45% of residents in the zip code where the center is located [9].

Between 4,500 and 5,000 outpatient visits are completed at this medical office annually. Patients are generally <30 years old (74.8%), non-white (52% African American and 9% multi-racial), and have Medicaid (49.5%); 8% have no health insurance.

### Data collection

Institutional Review Board approval was received for this project. Delivery of smoking cessation services was documented by a systematic review of medical records (for a further description on study design, please refer to Mahoney, et. al. 2014 [10].

A baseline chart review (n=261) was completed in 2012, along with a follow-up chart review in 2013 (n=268) which was approximately 6 months after completion of the educational sessions presented to enhance the knowledge and skills of office staff with regard to smoking cessation. A structured abstraction form was used to collect data on patient demographics, smoking status, and reason for visit. Medical charts for both reviews were available in paper format. Using a random starting point, every 10th chart was reviewed for the baseline review, and every 5th chart was reviewed for the post intervention review. Patients must have been identified as a smoker and have had an office visit within the prior 12 months to be eligible for each review.

### Independent variables

Chart reviews collected patient demographic information (gender, age, race/ethnicity), number of patient visits within the prior 12 months and selected medical data.

### Dependent variables

The main outcome variables included the proportion of smokers with an assessment of smoking status and provision of cessation support including the 5A’s (that is, advice to quit, assessment of readiness to quit, assistance with quitting including setting a quit date, provision of pharmacotherapy and counselling, and arranging for follow-up). For smokers who were not yet ready to quit, documentation of the 5Rs was assessed: whether relevance, risks, rewards, roadblocks and/or repetition were used as strategies to encourage smokers to quit.

### Statistical analysis

Data analyses was completed using SPSS Version 21 (© IBM, Armonk, NY), and included descriptive summaries and a comparison of baseline versus follow-up data using the chi-square statistic. Significance was established at p<0.05 without adjustment for multiple hypothesis testing.

## Results

### Demographic characteristics

As shown in Table 1, 48% of smokers were white and 38% were African-American; 9% were Hispanic. Mean age was 27 years and median age was 23 years; smokers ranged in age from 15–69 years old. In general, there were no differences in demographic variables between the pre-intervention and post-intervention chart review with the exception of Hispanic ethnicity which (p<0.001). That p-value reflects a decrease in the percentage of smokers with unknown data for ethnicity without a change in the proportion of smokers known to be of Hispanic ethnicity (p=0.19 based on Hispanic vs non-Hispanic/unknown dichotomy).

Most smokers had just a single office visit in the past 12 months. A majority of smokers (~94%) were still using tobacco based upon last recorded information. Smoking status was recorded as a vital sign for 75% of smokers at the baseline assessment and for 69% of smokers at the follow-up assessment (p=0.10).

### Smoking Cessation Services

Overall, 93% of smokers were asked about smoking status at their last visit based on the baseline and follow-up chart reviews. As shown in table 2, at baseline 40% of smokers were advised to quit smoking. This proportion dropped to 22% at the follow-up assessment (p<0.001). Significant but modest improvements were noted in the proportion of smokers who were assessed with regard to readiness to quit (3% baseline, 9% follow-up; p<0.003), however few smokers at baseline or follow-up were assessed for interest in setting a quit date or offered pharmacotherapy. The proportion of smokers who were counselled in the office increased from 12% at baseline to 41% at follow-up (p<0.001). Referral to the NYS smokers quit line increased from <1% at baseline to 8% at follow-up (p<0.001). When comparing “any assistance,” as defined more expansively as setting a quit date, or providing pharmacotherapy, or referring to smoking cessation program, or referring them to the NYS Quitline, an improvement was apparent (<1% baseline, 9% follow-up, p<0.001).

## Discussion

This study summarizes results from two medical chart audits performed to assess the impact of a series of 3 educational programs presented to medical office staff to encourage enhanced delivery of cessation services to smokers presenting for care at a women/s health centre. The educational sessions included information on the systematic identification of smokers by assessing smoking status as a vital sign, a review of evidence-based pharmacotherapy to support a quit attempt and use of motivational interviewing techniques to counsel smokers. While office staff reported that the educational sessions were useful, it is possible that additional sessions, including role playing with coaching and feedback, might have served to further reinforce practical approaches and skills for engaging patients in cessation. Also, providing more frequent periodic feedback on the extent of cessation services delivered may help to prioritize this activity.

Although the clinical practice guidelines for treating tobacco use and dependence [3] endorse the assessment of smoking as a vital sign, and this concept was emphasized at 2 of the 3 sessions, these interventions yielded no change; 75% of smokers at baseline had smoking status recorded as a vital sign compared to 69% at the follow-up assessment. The delivery of cessation assistance begins with the systematic identification of smokers which increases the likelihood that a clinical intervention will take place [3]. This office was effective at identifying the vast majority of smokers (93%) using strategies other than assessing smoking as a vital sign, including the collection of this information as part of the social history for each patient, however this approach is not systematic as recommended in clinical practice guidelines [3]. and may be overlooked by the treating clinician.

The medical chart review did identify areas with significant changes between baseline and follow-up: a decrease in the proportion of smokers being advised to quit (from 40% to 22%), an increase in the proportion of smokers assessed for readiness to quit (from 3% to 9%), an increase in the proportion of smokers who were counselled regarding a quit attempt increased (from 12% to 41%) and an increase in referral to the smokers quit line (from <1% to 8%). The decrease in the proportion of smokers advised by a clinician to quit smoking was unanticipated and may reflect limited documentation in the medical charts. The increase in the proportion of smokers assessed for readiness to quit was modest but may serve to establish a foundation for further improvements in the future.

The proportion of smokers who were counselled regarding a quit attempt was observed to have increased from 12% to 41%. This observation however, is not consistent with other data points. This change likely reflects a modification in the content of a paper based medical note used to document annual office visits to include a check box if the patient was “counselled to quit smoking”. As such, that check box would appear to be more consistent with providing “advice to quit”, rather than actual counselling support. In this regard, it seems likely that this reflects notations in the medical chart that a smoker was “advised” or “encouraged” to quit smoking rather than action-oriented counselling and/or motivational interviewing, as presented in one of the educational sessions. Also, the provision of pharmacotherapy as part of any quit attempt was quite low. It should be noted that the depth and breadth of counselling support provided to a smoker as part of a quit attempt may be difficulty to fully characterize based upon a chart review.

The rate of referral among smokers in this office to the state smokers quit line increased to 8% from a baseline of <1%. While this is a modest actual increase, it is a very large relative increase and is noteworthy as it may reflect a growing awareness of the value of quit lines to work cooperatively with medical offices to promote cessation. State quit lines have been shown to be effective at providing smokers with evidence-based approaches to support quitting including provision of free pharmacotherapy and telephone based counselling support [11].

For smokers who are not yet ready to quit, the Public Health Service guidelines for treating tobacco use and dependence [3]. Encourage clinicians to discuss relevance, risks, rewards, roadblocks and/or repetition (e.g., the 5 R’s) as strategies to encourage smokers to make a future quit attempt. Chart reviews identified that the 5 R’s were discussed with patients during only 2% of visits at baseline and only 1% of visits based on the follow-up assessment. This may reflect limited documentation of such conversation or the need to more clearly communicate to the patient the importance of cessation and willingness to provide assistance.

Compared to the demographics of the surrounding city and county, patients at this women’s health centre included a disproportionate number of non-whites, mostly African Americans, likely reflecting both the local community demographics and socioeconomic challenges among the patient base who sought medical care at this women’s health center. Smoking rates among Hispanics (12.5%) and Asians (10.7%) are lower than among African-Americans (18.1%), whites (19.7%) and American Indians/Alaska Natives (21.8%) and among persons reporting multiple races (26.1%) [1]. Also, low socioeconomic status (SES) is linked with higher smoking rates as well as poorer health outcomes [12]. About one-half of patients at this office had Medicaid insurance and another 8% had no health insurance. While the neighbourhood surrounding this medical office is socioeconomically heterogeneous, and 30% of individuals were below the poverty level [9]. The enhanced array of services available in patient centered medical homes may help to mobilize the necessary resources to better address the unique social and economic barriers to accessing smoking cessation among underserved populations [10].

Strengths of this study include the use of a quasi-experimental design to assess intervention impact, and a comprehensive review of a sample of medical records. Limitations includes the reliance upon information as recorded in medical charts; any additional counselling or advice offered to patients that may have occurred and was not recorded on the medical chart was not included. While this office continues to use a paper-based records system conversion to an electronic-based medical records (EMR) system is imminent.

Overall, these results suggest a modest impact in the delivery of smoking cessation services to smokers at a women’s health center resulting from a series of 3 education programs presented to office staff. The limited changes may reflect challenges in making changes in process and practice at this office, competing demands of clinical care, a lack of impact from the educational interventions and/or some combination of factors. Implementation of the EMR will likely aid in the systematic identification of smokers as well as enhance the ability to prompt providers to encourage and support quit attempts using the information acquired in the series of educational program.

## Conclusion

This effort was useful in serving to focus attention on smoking as an important health behaviour and may lead to on-going quality improvement/quality assurance activities on this topic in this office. An unanticipated outcome was the adoption of a smoke-free policy at this medical office. Moreover, it serves as an example of partnership between a community-based medical office and an academic health center coming together to promote smoking cessation.

## Figures and Tables

**Table 1:** Selected demographic and tobacco use variables, Women’s Health Centre, baseline and follow-up assessment

Variables		baseline	follow-up	
		n (%)	n (%)	p-value
**Gender**	Female	261 (100%)	268 (100%)	
**Race/Ethnicity**	White	131 (50%)	124 (46%)	0.58
	African American	99 (38%)	103 (39%)	
	Other/Multi Racial	23 (9%)	33 (12%)	
	Not Available/Unknown	8 (3%)	8 (3%)	
**Hispanic Ethnicity**	Hispanic/Latino	18 (7%)	27 (10%)	<0.001
	Non-Hispanic	110 (42%)	179 (67%)	
	unknown	133 (51%)	62 (23%)	
**Age (in years)**	Mean	27.2	26.7	0.42
	Median	25	25	0.53
	Range	15–59	15–68	
**Office visits in past year**	1	165 (63%)	149 (56%)	0.15
	2	53 (20%)	77 (29%)	
	3	26 (10%)	24 (9%)	
	4 or more	17 (6%)	18 (6%)	
**Last recorded smoking status?**	Current	244 (93%)	255 (95%)	0.86
	Former	17 (7%)	12 (5%)	
**Smoking recorded as a vital sign?**	Yes	196 (75%)	184 (69%)	0.1
	No	65 (25%)	84 (31%)	

**Table 2:** Delivery of Smoking Cessation Support, Women’s Health Center, baseline and follow-up assessment

Variables		baseline	follow-up	p-value
		n (%)	n (%)	
Smoking status assessed at last office visit?	Yes	242 (93%)	250 (93%)	0.8
	No	19 (7%)	18 (7%)	
Was patient advised to quit?	Yes	103 (40%)	59 (22%)	<0.001
	No	158 (60%)	209 (78%)	
Was readiness to quit assessed?	Yes	8 (3%)	25 (9%)	<0.003
	No	253 (97%)	243 (91%)	
Was quit date established?	Yes	0	2 (1%)	0.16
	No	261 (100%)	266 (99%)	
Was pharmacotherapy offered?	Yes	0	1 (1%)	0.32
	No	261 (100%)	267 (99%)	
Was patient counseled in office?	Yes	32 (12%)	109 (41%)	<0.001
	No	229 (88%)	159 (59%)	
Was patient referred to state smokers quit line?	Yes	1 (<1%)	22 (8%)	<0.001
	No	260 (99%)	246 (92%)	
Was smoker provided with “any assistance”?*	Yes	1 (12%)	24 (9%)	<0.001
	No	260 (88%)	244 (91%)	

Baseline=pre-intervention; follow-up=post-intervention. “any assistance” was defined as setting a quit date with patient, providing a script for pharmacotherapy, offering pharmacotherapy, referring to a cessation program or referring to the state smokers quit line.

## References

[1] Agaku IT, King BA, Dube SR, Centers for Disease Control and Prevention (CDC) (2014). Current cigarette smoking among adults - United States, 2005–2012. MMWR Morb Mortal Wkly Rep.

[2] US Department of Health and Human Services (2014). The health consequences of smoking 50 years of progress: A report of the surgeon general.

[3] Fiore MC (2000). US public health service clinical practice guideline: treating tobacco use and dependence. Respir Care.

[4] Jha P, Ramasundarahettige C, Landsman V, Rostron B, Thun M (2013). 21st-century hazards of smoking and benefits of cessation in the United States. N Engl J Med.

[5] Murphy JM, Mahoney MC, Cummings KM, Hyland AJ, Lawvere S (2005). A randomized trial to promote pharmacotherapy use and smoking cessation in a Medicaid population (United States). See comment in PubMed Commons below. Cancer Causes Control.

[6] Centers for Disease Control and Prevention (CDC) (2011). Quitting smoking among adults--United States, 2001–2010. MMWR Morb Mortal Wkly Rep.

[7] American Cancer Society (2012). Cancer prevention & early Detection Facts & figures 2012.

[8] Thomas GS (2009). Buffalo names third-poorest city in US.

[9] U S Census Bureau (2010). Buffalo New York.

[10] Mahoney MC, Masucci Twarozek A, Saad-Harfouche F, Widman C, Erwin DO (2014). Assessing the delivery of cessation services to smokers in urban, safety-net clinics. J Community Health.

[11] Ossip-Klein DJ, McIntosh S (2003). Quitlines in North America: evidence base and applications. Am J Med Sci.

[12] Cokkinides VE, Halpern MT, Barbeau EM, Ward E, Thun MJ (2008). Racial and ethnic disparities in smoking-cessation interventions: analysis of the 2005 National Health Interview Survey. Am J Prev Med.

